# Aberrant functional connectivity in anterior cingulate gyrus subregions in migraine without aura patients

**DOI:** 10.3389/fneur.2024.1412117

**Published:** 2024-07-17

**Authors:** Jinming Cheng, Yan Li, Keyang Chen, Yungang Cao, Kun Liu, Xi Zhang, Xiaoyuan Wu, Zhihong Wang, Xiaozheng Liu, Litao Li

**Affiliations:** ^1^Department of Neurology of the Hebei Medical University, Shijiazhuang, Hebei, China; ^2^Department of Neurology of the Hebei General Hospital, Shijiazhuang, Hebei, China; ^3^Department of Neurology of the Second Affiliated Hospital and Yuying Children’s Hospital, Wenzhou Medical University, Wenzhou, Zhejiang, China; ^4^Department of Radiology of the Second Affiliated Hospital and Yuying Children’s Hospital, Wenzhou Medical University, Wenzhou, Zhejiang, China; ^5^Department of Neurology of Xingtai People’s Hospital, Xingtai, Hebei, China; ^6^Department of Neurology of the Second Affiliated Hospital, Hebei Medical University, Shijiazhuang, Hebei, China

**Keywords:** migraine without aura, anterior cingulate gyrus, functional connectivity, functional magnetic resonance imaging, resting state

## Abstract

**Background:**

The anterior cingulate gyrus (ACG) is an important regulatory region for pain-related information. However, the ACG is composed of subregions with different functions. The mechanisms underlying the brain networks of different subregions of the ACG in patients with migraine without aura (MwoA) are currently unclear.

**Methods:**

In the current study, resting-state functional magnetic resonance imaging (rsfMRI) and functional connectivity (FC) were used to investigate the functional characteristics of ACG subregions in MwoA patients. The study included 17 healthy volunteers and 28 MwoA patients. The FC calculation was based on rsfMRI data from a 3 T MRI scanner. The brain networks of the ACG subregions were compared using a general linear model to see if there were any differences between the two groups. Spearman correlation analysis was used to examine the correlation between FC values in abnormal brain regions and clinical variables.

**Results:**

Compared with healthy subjects, MwoA patients showed decreased FC between left subgenual ACG and left middle cingulate gyrus and right middle temporal gyrus. Meanwhile, MwoA patients also showed increased FC between pregenual ACG and right angular gyrus and increased FC between right pregenual ACG and right superior occipital gyrus. The FC values between pregenual ACG and right superior occipital gyrus were significantly positively correlated with the visual analogue scale.

**Conclusion:**

Disturbances of FC between ACG subregions and default model network and visual cortex may play a key role in neuropathological features, perception and affection of MwoA. The current study provides further insights into the complex scenario of MwoA mechanisms.

## 1 Introduction

Migraine is a neurological disorder characterised by paroxysmal unilateral throbbing headache and autonomic nervous system dysfunction. The prevalence of migraine is 10–15% of the population and represents a significant personal and social burden (1). Migraine without aura (MwoA) is one of the most common types of migraine and is characterised by unilateral or bilateral frontotemporal pain with recurrent throbbing episodes. The headaches may be accompanied by nausea, vomiting, scalp tenderness and other unpleasant symptoms, and if the attacks are frequent, they can seriously interfere with daily life and work (2).

The anterior cingulate gyrus (ACG) is part of the limbic system and a component of the medial nociceptive system (3). A large number of functional magnetic resonance imaging (fMRI) studies have shown functional abnormalities in the ACG in migraineurs, such as amplitude of low-frequency fluctuations, regional homogeneity, and functional connectivity (4). A meta-analysis shows an association between reduced grey matter density in the right ACG and migraine attack frequency (5). Improvement in migraine frequency, intensity and disability in migraineurs after 3 months of long-term treatment is associated with increased Gamma-aminobutyric acid + macromolecules (GABA+) levels in the ACG (6). After thermal pain stimulation, the anterior cingulate gyrus was significantly activated in migraineurs compared to healthy controls (HCs) (7).

However, the ACG is composed of cytoarchitecturally distinct subdomains that serve multiple functions. According to the different corresponding functions, the ACG can be divided into different subregions, including the pregenual anterior cingulate gyrus (pgACG), the subgenual anterior cingulate gyrus (sgACG) and the supracallosal anterior cingulate gyrus (srACG) (8). The pgACG was more strongly connected to the default mode network (DMN) in patients with low back pain, and there was a positive correlation between clinical pain and the strength of the DMN connection (9). After receiving noxious and non-noxious hot and cold stimuli and finger movement tasks, healthy subjects showed different partial activation of the ACG. Non-noxious heat stimulation activated the anterior part of the ACG, noxious heat-related activation activated the ventral part of the ACG and motor-related activation activated the posterior part of the ACG (10).

The above studies showed that there were functional abnormalities with different characteristics in the ACG subregions of migraine patients, especially functional network abnormalities (4, 9). Functional connectivity, defined as the time-dependent pattern of neuronal activation in anatomically separated brain regions, had been widely used to reveal the overall organisation of functional communication in brain networks (4, 9). To our knowledge, brain network characterisation of ACG subregions in migraineurs is still lacking. In the current study, we hypothesised that different subregions of the ACG in MwoA patients have their own specific brain network patterns. We used resting state fMRI (rsfMRI) and the functional connectivity (FC) method to investigate changes in the brain networks of different subregions of the ACG in MwoA patients. We hypothesised that there is abnormal functional connectivity between ACG subregions and visual, cognitively relevant brain regions in migraineurs compared to healthy controls.

## 2 Methods

### 2.1 Participants

Between March 2014 and October 2014, 28 right-handed MwoA patients and 17 age- and sex-matched right-handed HCs were recruited for this study. The diagnosis of definite MwoA was made according to the criteria of the International Headache Society (11) by two neurologists specialising in headache disorders who were blinded to the MRI and neuropsychological findings. The following requirements had to be met by the patients: (a) the right hand must be used as the usual hand; (b) the patient must be over the age of 18; (c) the patient must have experienced migraine symptoms for more than 6 months; (d) the patient must have experienced headache attacks at least twice a month (as confirmed by the patient’s self-reports prior to the study); and (e) patients had no headache attacks during the fMRI scan and had not taken any acute migraine medication for at least 3 days prior to the scan. Exclusion criteria included headache attacks in the 3 days prior to the scan, on the day of the scan, or in the 3 days after the scan; history of substance abuse or prophylactic drug use; psychotic disorder; contraindications to MRI scanning; female subjects who were pregnant or menstruating. The control subjects were recruited from the local community and had no history of neurological disease. They did not have migraines or headaches and were not taking any medication. This study was approved by our university’s institutional review board. All participants gave written informed consent before undergoing the procedure.

### 2.2 MRI scan

Images were acquired on a 3.0 Tesla magnetic resonance imaging scanner (Achieva X-series, Philips Medical, Best, the Netherlands). Functional images were acquired axially using a gradient-echo planar imaging sequence as follows: repetition time (TR) = 2,000 ms; echo time (TE) = 30 ms; slice = 35; thickness = 4 mm; gap = 0 mm; field of view (FOV) = 240 mm × 240 mm; acquisition matrix = 80 × 80; flip angle (FA) = 90°. fMRI sequences took 8 min. We instructed patients to remain still and close their eyes during image acquisition.

### 2.3 Data processing

Data preprocessing was performed using SPM12^1^ and the Resting State fMRI Data Analysis Toolkit+V1.25 (RESTplus V1.25)^2^ (12). The preprocessing steps were as follows: the first 10 volumes were discarded to allow the signal to reach equilibrium, corrected for slice time and head motion, spatially normalised to Montreal Neurological Institute (MNI) space using EPI template, and resampled to a resolution of 3 × 3 × 3 mm^3^, smoothed with an isotropic Gaussian kernel (full width at half maximum = 6 mm) to reduce registration errors, linear trend removal and temporal band-pass filtering (0.01–0.08 Hz) to remove high-frequency noise and physiological signals. Finally, interference was further removed from the resulting images by regressing out the head motion parameters (Friston 24 model), cerebrospinal fluid signal and white matter signal. Subjects with head movements greater than 2.0 mm translational or 2.0° rotational in any direction were excluded.

### 2.4 Resting-state functional connectivity analysis

**Figure 1:**
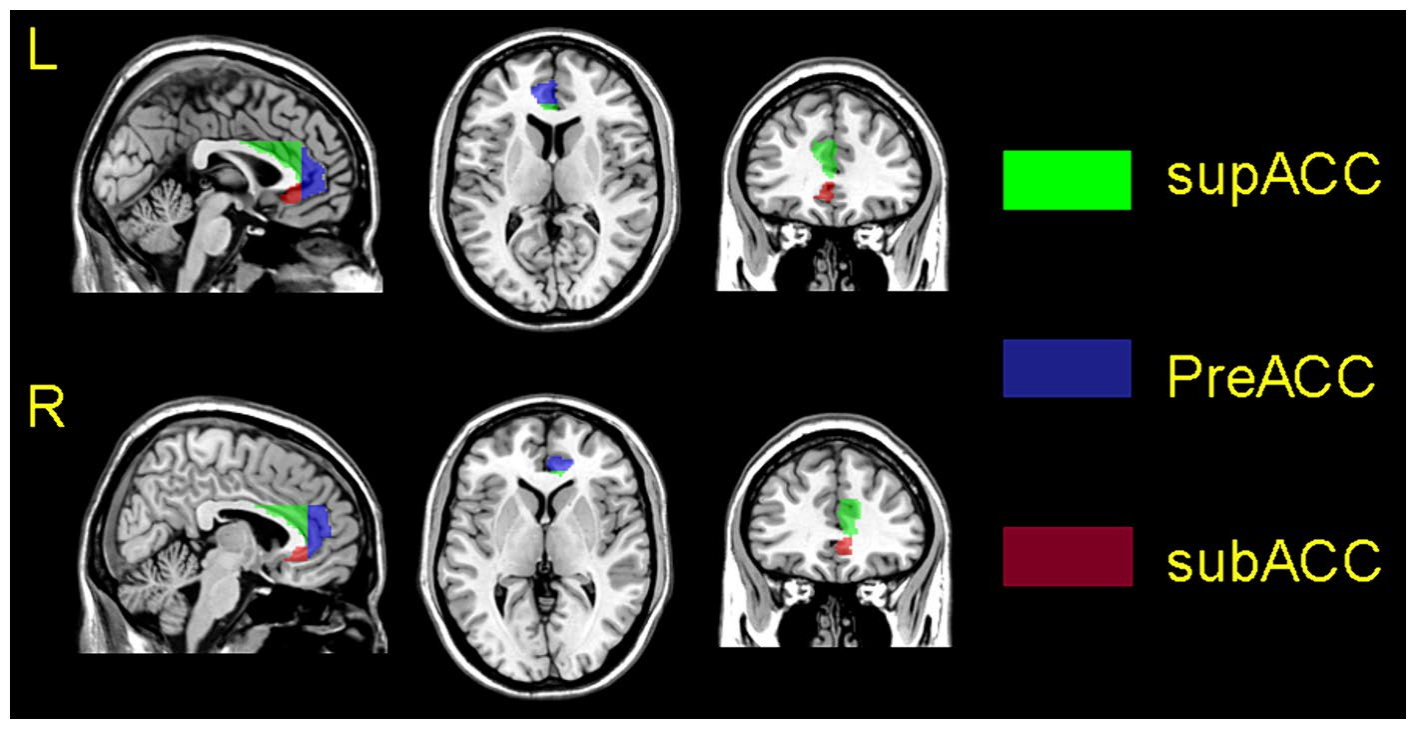
Schematic representation of the positioning of the ACG subregions, superimposed on the ch2 template. The upper picture shows the left side of the ACG, the bottom picture shows the right side of the ACG. The subgenual anterior cingulate gyrus is marked in orange; the pregenual anterior cingulate gyrus is marked in blue, and supracallosal anterior cingulate gyrus is marked in green.

Using an atlas-based approach, six individual ACG subregion seeds (three per hemisphere) were generated for each participant in the AAL3 template (13) (Figure 1). Pearson correlation coefficients were calculated between mean seed time series and other whole-brain voxel time series. The resulting values were transformed to z-values using Fisher’s z-transformation to improve normality (14).

### 2.5 Statistical analysis

The Mann–Whitney U-test was used to compare the age and education of the two groups to determine whether or not there was a difference in applied mathematics. Chi-square was used to compare the gender composition of the two groups.

We performed statistical analysis using RESTplus V1.25. The normal distribution of the fMRI signal was determined using the Jarque-Bera test. We used a one-sample t-test to observe the functional connectivity mapping of the ACG subregions of the two groups. A general linear model was performed on individual FC maps that were voxel-by-voxel-normalised between the two groups. To reduce the influence of confounding variables in the statistical analysis, we regressed the mean relative change in head movement, age, and sex as covariates in the statistical analysis. For multiple comparisons, the resulting statistical map was set to *p* < 0.05 (AlphaSim corrected for multiple comparisons, with individual combined *p*-values for voxels <0.001 with cluster size > 16 voxels). To investigate the association between FC value and clinical outcome in MwoA patients, a spearman correlation between the Z value of abnormal brain regions and clinical outcome of MwoA patients was performed in a voxel system. The statistical threshold was set at *p* < 0.05.

## 3 Results

### 3.1 Neuropsychological results

There were no significant differences in age (*t* = −0.270, *p* = 0.7873), gender distribution (*χ2* = 2, *p* = 0.3431), or years of education (*t* = 0.973, *p* = 0.2030) between the two groups. Details of the demographic data and associated tests are shown in Table 1.

**Table 1:** Demographics and neuropsychological data.

	MwoA	Median	Q25	Q75	IQR	HCs	t/χ^2^	*p*-value
Gender, n (M/F)	28(22/6)	-				17(11/6)	2	0.3431
Age, years	35.6 ± 9.9	38.5	27.5	43	15.5	36.5 ± 8.9	−0.270	0.7873
Education (years)	13 ± 4	15	9	16	7	15 ± 4	0.973	0.2030
Duration (years)	9 ± 7	6.75	3	14.5	11.5	-	-	-
Frequency (d/m)	4.7 ± 7.7	2.5	1.62	3.12	1.6	-	-	-
VAS score	6.85 ± 1.3	6.75	5.25	7.25	2.25	-	-	-

Data represent mean ± SD. Data were analysed using independent-samples t-tests. MwoA, Migraine without aura; HCs, Healthy controls; d/m, day per month; VAS, visual analogue scale; Q25, 25% quantile; Q75, 75% quantile; IQR, interquartile range.

### 3.2 Altered FC of ACG subregions in MwoA patients

**Figure 2:**
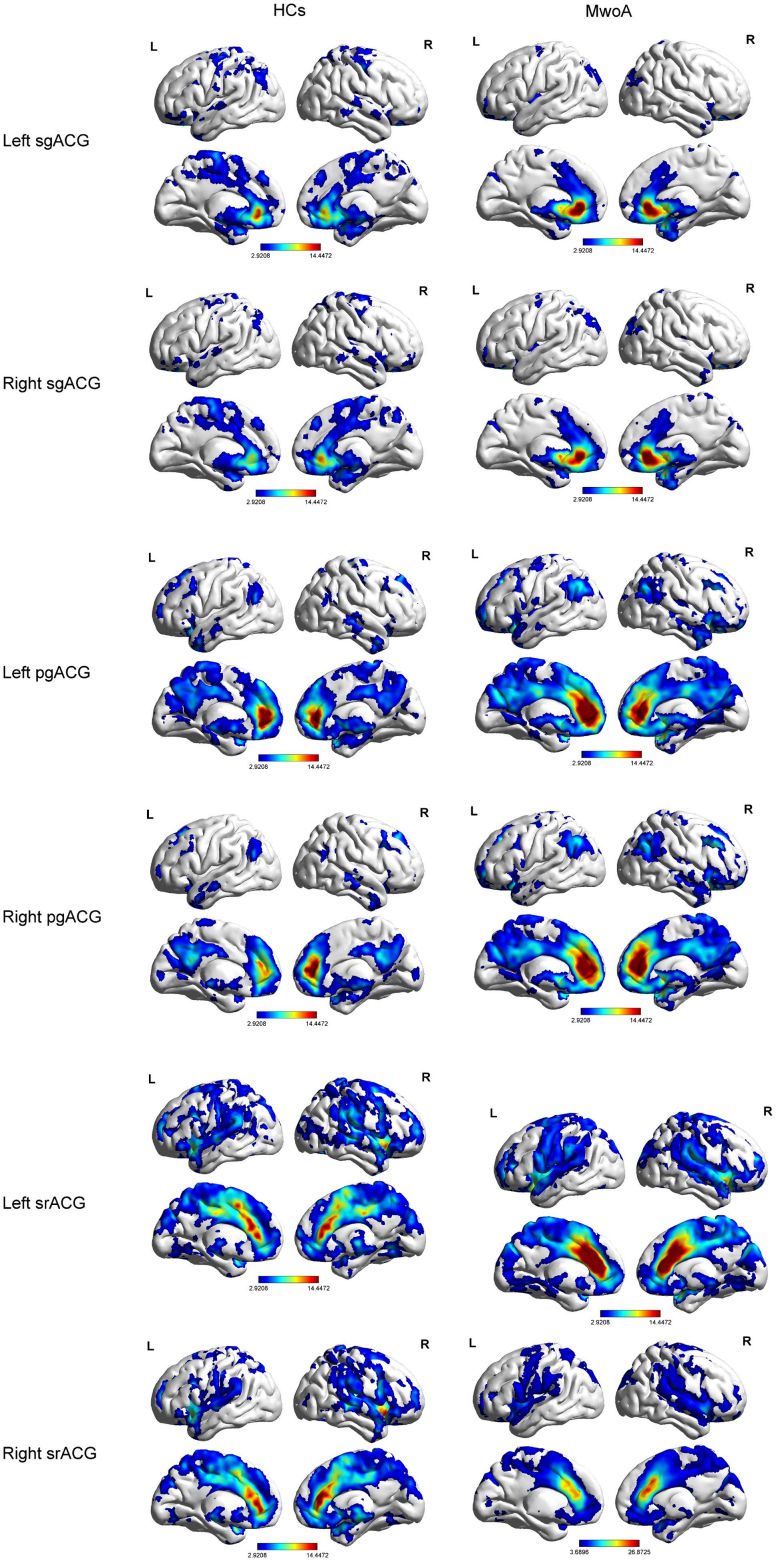
Functional connection mapping of the anterior cingulate subregions. pgACG, pregenual anterior cingulate gyrus; sgACG, subgenual anterior cingulate gyrus; srACG, supracallosal anterior cingulate gyrus.

The results of the one-sample t-test revealed, compared to HCs, MwoA patients showed reduced functional connectivity between the subgenual ACG with the default mode network, ventral attention network, and somatomotor network. MwoA patients showed enhanced functional connectivity between the pregenual ACG with the default mode network and frontoparietal network. MwoA patients showed enhanced functional connectivity between the supracallosal ACG with the default mode network (Figure 2).

**Figure 3:**
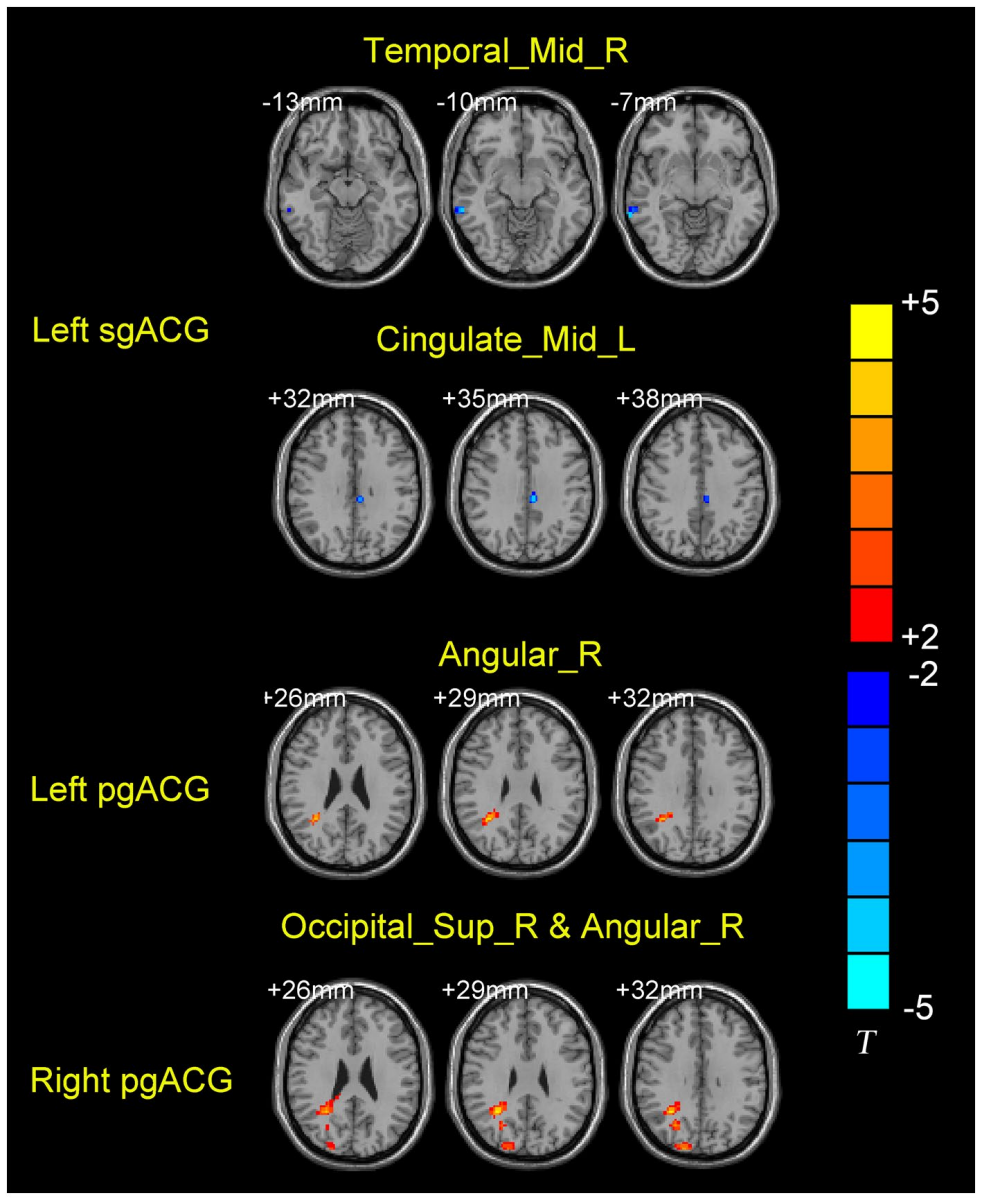
Brain regions with significantly different FC values with ACG subregions in the MwoA group compared with the HCs group.

Compared to HCs, MwoA patients showed decreased FC between left subgenual ACG and left middle cingulate gyrus (MCG) and right middle temporal gyrus (MTG). Meanwhile, MwoA patients also showed increased FC between pregenual ACG and right angular gyrus (AG) and increased FC between right pregenual ACG and right superior occipital gyrus (SOG; Figure 3; Table 2).

**Table 2:** Brain regions with significantly different FC values with ACG subregions in the MwoA group compared with the HCs group.

Brain regions	Voxels	BA	MNI coordinates			*T* value	*p-*value
			x	y	z		
**Left sgACG**							
Temporal_Mid_R	23	21	69	−42	−6	−4.6144	<0.001
Cingulum_Mid_L	19	23	−6	−30	36	−4.5804	<0.001
**Left pgACG**							
Angular_R	48	39	40	−53	28	5.1178	<0.001
**Right pgACG**							
Angular_R	79	40	33	−49	40	5.5695	<0.001
Occipital_Sup_R	33	19	18	−90	33	4.5178	<0.001

MwoA, Migraine without aura; HCs, Healthy controls; MNI, Montreal Neurological Institute; BA, Brodmann area.

### 3.3 Relationships between FC values and clinical variables

**Figure 4:**
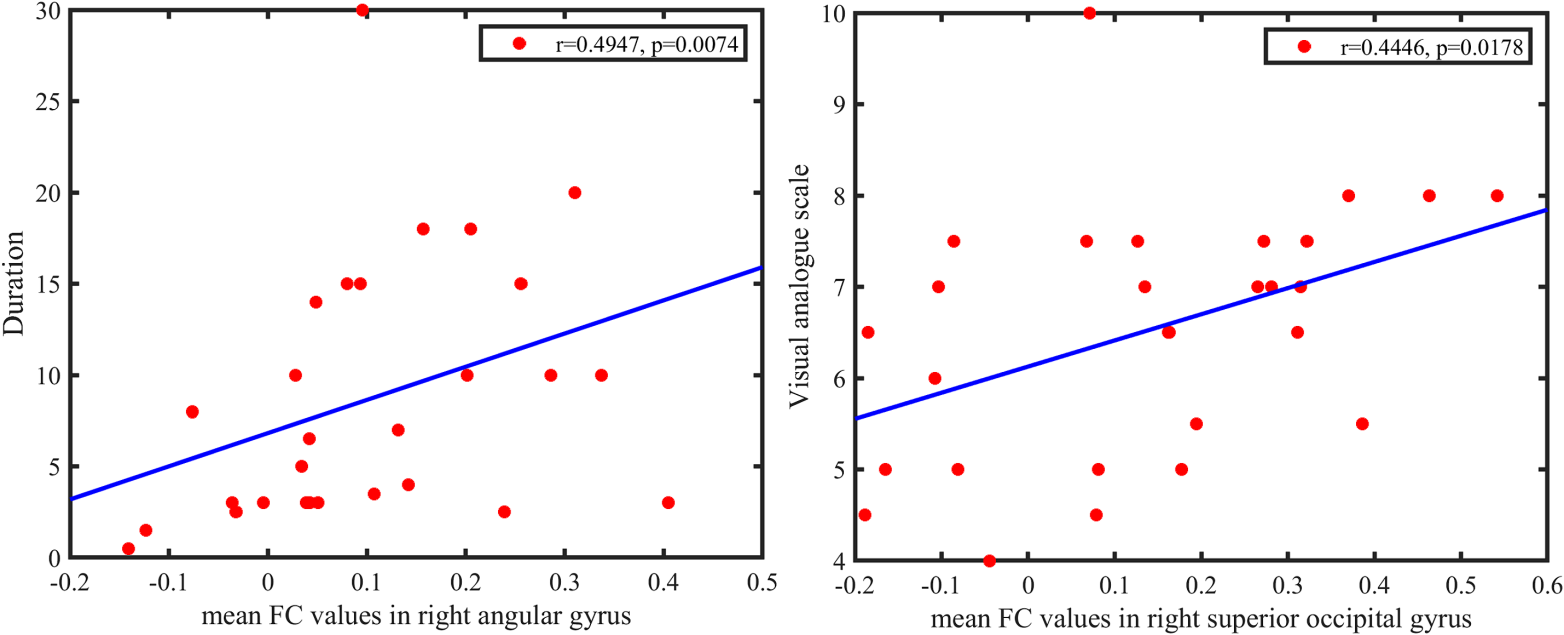
Top: Functional connectivity values between pgACG and right SOG are significantly positively correlated with visual analogue scale. Bottom: FC values between pgACG and right AG are weakly positively correlated with disease duration. pgACG, pregenual ACG; SOG, superior occipital gyrus; AG, angular gyrus.

The FC values between pgACG and right SOG were significantly positively correlated with the visual analogue scale (r = 0.4446, *p* = 0.0178). The FC values between pgACG and right AG were positively correlated with disease duration (r = 0.4947, *p* = 0.0074; Figure 4).

## 4 Discussion

In the current study, we investigated changes in the brain networks of the ACG subregions in MwoA patients. Compared to HCs, MwoA patients showed decreased FC between left subgenual ACG and left middle cingulate gyrus and right middle temporal gyrus. Meanwhile, MwoA patients also showed increased FC between pregenual ACG and right angular and increased FC between right pregenual ACG and right occipital gyrus. Abnormal brain regions are mainly located in the default mode network (DMN) and the visual cortex.

Our results show an unusual FC between the left sgACG and the left MCG and right MTG. The sgACG plays an important role in pain-related emotional behaviour. After active transcranial direct current stimulation, patients with chronic neuropathic pain showed a significant decrease in pain scores and increased metabolism in the sgACG and insula (15). Activation of sgACG in chronic cluster headache patients using transcranial direct current stimulation improved their clinical symptoms (16). The Mulligan manoeuvre can improve pain levels by modulating the function of the frontal lobe and middle temporal gyrus related brain regions in patients with cervicogenic headache, and has a moderating effect on pain-related negative emotions (17). Meta-analysis showed that pain-related anxiety is characterised by neural activation in the inferior frontal gyrus, medial superior frontal gyrus, postcentral gyrus, MTG, parieto-occipital sulcus and striatum (18). Compared with healthy controls, migraineurs showed increased regional homogeneity scores in the bilateral thalamus, right insula and right MTG (19). Our findings suggest that MwoA patients have abnormalities in emotion-related brain networks.

Our results show an increased FC between the pgACG and the AG and right SOG. PgACG segregate representations of rewarding, positively affective (pleasant) stimuli (8). Painful touch can activate pregenual ACGs in HCs (20). A small increase in 5-Hydroxytryptamine (5-HT) levels leads to increased activation of the pgACG, suggesting that activation of the pgACG may also be increased during migraine attacks, a process that may be associated with sudden increases in 5-HT levels (21). Compared with the HCs, the neurovascular coupling function of SOG and AG in patients with chronic migraine was abnormal, and it was negatively correlated with headache frequency and positively correlated with health status (22). The angular gyrus is the visual language centre and impaired function can lead to dyslexia (23). Migraine is a factor in aphasia (24). The occipital lobe is the main brain area for visual perception and is also responsible for visual language processing (25). HCs showed increased activity in the left SOG and left AG during artificial grammar learning (26). MwoA patients also showed abnormal efficient connections from the middle occipital gyrus to the right periaqueductal grey (27). Our results suggest a compensatory mechanism in visual language processing in MwoA patients.

The abnormal brain areas suggested by our results are mostly located in the DMN. When pain persists beyond healing and becomes chronic, pain-related somatosensory cortical activity may become functionally linked to the DMN of self-representation, i.e., it becomes an intrinsic part of self-perception (28). The default mode network has been proposed as a biomarker for several chronic pain conditions. The default mode network FC is influenced by negative emotions and may be related to the emotional dimension of pain in patients with chronic pain (29). MwoA patients also show reduced FC in the prefrontal and temporal regions of the DMN (30). It is not only the pain pathway but also migraine that leads to emotional changes in MwoA patients, which has a broader impact on brain function in MwoA patients.

Several limitations should be considered when interpreting the current results. First, multi-site and large sample data are needed to validate the reliability of the results of this study. Second, because the current study used a cross-sectional design, it investigated differences in the ACG brain network between MwoA and HCs. However, whether and how these ACG brain network abnormalities exist in the onset and development of MwoA needs to be further observed in longitudinal studies in the future. Third, diffusion imaging can be used to explore the mechanisms of white matter microstructure in the ACG subregions. Finally, our results revealed abnormalities in brain regions related to mood and vision, but we did not obtain clinical scales for mood and vision in this study, and we were unable to further explore the neural mechanisms of the brain regions associated with MwoA patients.

## 5 Conclusion

We investigated the FC of ACG subregions in MwoA patients using resting-state functional MRI and the FC method. Compared to HCs, the differential brain regions in MwoA patients are mainly located in the DMN and visual cortex. Our study extends our understanding of the brain network patterns in the ACG subregions of MwoA patients and helps us to further understand the underlying neural mechanisms in MwoA patients.

## Data availability statement

The raw data supporting the conclusions of this article will be made available by the authors, without undue reservation.

## Ethics statement

The studies involving humans were approved by the Second Affiliated Hospital, Hebei Medical University. The studies were conducted in accordance with the local legislation and institutional requirements. The participants provided their written informed consent to participate in this study.

## Author contributions

JC: Data curation, Formal analysis, Funding acquisition, Resources, Writing – original draft, Writing – review & editing. YL: Data curation, Formal analysis, Methodology, Resources, Validation, Visualization, Writing – review & editing. KC: Data curation, Formal analysis, Resources, Validation, Writing – review & editing. YC: Data curation, Formal analysis, Investigation, Writing – review & editing. KL: Methodology, Resources, Validation, Visualization, Writing – review & editing. XZ: Data curation, Formal analysis, Resources, Writing – review & editing. XW: Data curation, Investigation, Resources, Writing – review & editing. ZW: Project administration, Validation, Visualization, Writing – review & editing. XL: Methodology, Project administration, Software, Supervision, Writing – review & editing. LL: Conceptualization, Project administration, Writing – review & editing.

## Footnotes

^1^ http://www.fil.ion.ucl.ac.uk/spm

^2^ http://www.restfmri.net
